
JOURNAL INFORMATION
==============================
NLM Title Abbreviation: J Comp Med Vet Arch
NLM Journal ID: 17840220R

The Journal of Comparative Medicine and Veterinary Archives

Publisher: Biomedical Journal Digitization Project

ARTICLE INFORMATION
==============================
PMCID: PMC9346715
PMID: 36393588
Article version: 1
Subjects: Among the Colleges

Among the Colleges

Print publication date: 1900 Oct
Volume: 21
Issue: 10
First page: 648
Last page: 648
License: This work is free of known copyright restrictions. For more information, please see PMC Back Issue Digitization.
License URL: https://creativecommons.org/publicdomain/mark/1.0/

==============================

Coroner Porter, of Newcastle, never worries about inquests when Association meetings call him from home.

Member Irons, of Erie, was one of the strong advocates of the law passed in 1893 regulating the licensing of stallions.

### AMONG THE COLLEGES.

United States College of Veterinary Surgeons. In the new catalogue for 1900–1901 the only change noted is that in the professor of general pathology, J. B. Nichols, M.D., taking the place of Dr. Charles F. Dawson. The Chair of Professor of Anatomy remains vacant.

Chicago Veterinary College. Catalogue for 1900–1901 announces changes in Faculty as follows: As the Professor of Dental Surgery, James Robertson, M.D.C., takes the place of G. E. Totten, M.D.C.; John D. Robertson, M.D., has taken the place of Edson B. Fowler, A.B., M.D., as Professor of Chemistry and Physics; O. H. Leonhart, M.D.C., Assistant to the Chair of Anatomy, in place of B. W. Murphy, M.D.C.

McKillip Veterinary College. Catalogue 1900–1901. Very few changes noticed. E. Merillat, A.B., M.D.V., takes the place of F. Schoenleber, M.S.A., D.V.S., as Dean and Professor of Anatomy and Histology; he also takes the place of S. G. Burkholder, M.D., D.V.S., as Clinician in Meat-inspection. Samuel E. Huntsberger, M.D.V., takes the place of J. B. Boomer, M.D.V., as Professor of Materia Medica and Pharmacy and on the Practicing Staff and as Laboratory Instructor. Added to the list of Professors Emeriti: F. S. Schoenleber, M.S.A., D.V.S.; J. B. Boomer, M.D.V., and Olof Schwartzkopf, V.M.

Ontario Veterinary College. The 1900 and 1901 catalogue makes no change in the staff of instructors. Several new works have been added to the list of text-books. The use of sectional frozen specimens of the entire horse, sheep, and other animals is now a very practical form of instruction adopted in this school.

**FOR SALE. BARGAIN.**

A rare chance for a Veterinarian to secure a good established practice of twelve years in a town of 2500 population in Western New York. Fine farming locality, no competition nearer than nine miles. Also, in connection with above practice, a first-class horse-shoeing trade, averaging $15.00 per day year round. Good reasons for selling; terms easy. Do not write unless you mean business and have $500, then address

JOURNAL OFFICE.
